# Prehospital Management of Pediatric Behavioral Health Emergencies: A Scoping Review

**DOI:** 10.7759/cureus.38840

**Published:** 2023-05-10

**Authors:** Elizabeth V Zorovich, Kathryn Kothari, Kathleen Adelgais, Rachael Alter, Lia Mojica, Aaron Salinas, Marc Auerbach, Carrie Adams, Jennifer Fishe

**Affiliations:** 1 Pediatric Emergency Medicine, University of Florida College of Medicine – Jacksonville, Jacksonville, USA; 2 Pediatric Emergency Medicine, Baylor College of Medicine, Houston, USA; 3 Pediatric Emergency Medicine, University of Colorado School of Medicine, Aurora, USA; 4 Emergency Medicine Services, Emergency Medicine Services for Children Innovation and Improvement Center, Austin, USA; 5 Pediatric Emergency Medicine, Medical College of Wisconsin, Milwaukee, USA; 6 Emergency Medicine Services, University of Texas Rio Grande Valley, Edinburg, USA; 7 Department of Pediatrics, Section of Pediatric Emergency Medicine, Yale School of Medicine, New Haven, USA; 8 Borland Library, University of Florida College of Medicine – Jacksonville, Jacksonville, USA

**Keywords:** behavioral health emergencies, emergency medical services, pediatric behavioral health emergency, pediatric emergency medicine, prehospital management

## Abstract

Pediatric behavioral health emergencies (BHE) are increasing in prevalence, yet there are no evidence-based guidelines or protocols for prehospital management. The primary objective of this scoping review is to identify prehospital-specific pediatric BHE research and publicly available emergency medical services (EMS) protocols for pediatric BHE. Secondary objectives include identifying the next priorities for research and EMS protocol considerations for children with neurodevelopmental conditions.

This is a scoping review comprised of a research literature search for publications from 2012-2022 and an internet search for publicly available EMS protocols from the United States. Included publications contain data on the epidemiology of pediatric BHE or describe prehospital management of pediatric BHE. EMS protocols were included if they had advisements specific to pediatric BHE.

A total of 50 research publications and EMS protocols from 43 states were screened. Seven publications and four protocols were included in this study. Research studies indicated an increase in pediatric BHE over the last decade, but few papers discuss current prehospital management (n=4). Two EMS protocols were specific to pediatric BHE or pediatric agitation, and the other two EMS protocols focused on adult populations with integrated pediatric recommendations. All four EMS protocols encouraged nonpharmaceutical interventions prior to the use of pharmacologic restraints.

Although there is a substantial rise in pediatric BHE, there is sparse research data and clinical EMS protocols to support best practices for prehospital pediatric BHE management. This scoping review identifies important future research aims to inform best practices for the prehospital management of pediatric BHE.

## Introduction and background

Over the past decade, there has been a steady rise in the incidence of pediatric behavioral health emergencies (BHE) in the United States (US). From 2007 to 2016, the number of pediatric patients seen for emergency department (ED) visits for deliberate self-harm increased by 329%, and visits for all pediatric mental health disorders rose by 60% [1]. In response to this increase, the American Academy of Pediatrics, American Academy of Children and Adolescent Psychiatry, and Children's Hospital Association declared a "National State of Emergency in Children's Mental Health" in 2021. The associations emphasized the effects of the COVID-19 pandemic in further exacerbating the pre-existing crisis in child and adolescent mental health in their declaration. This declaration calls to identify strategies and innovations using state, local, and national approaches to improve the access to and quality of care across the continuum of mental health promotion, prevention, and treatment for all children [2].

The rise in pediatric BHE is a concern for all health professionals, including prehospital emergency medical services (EMS). Every year in the US, EMS clinicians treat one to two million pediatric patients. They also serve as the first point of contact for many children with BHE [3]. Among EMS agencies utilizing ImageTrend™ software, a national EMS electronic medical record vendor, 11% of the 1,046,897 pediatric incidents in the US from 2018-2021 were BHE [4]. According to National EMS Information System Data (NEMSIS), mental health and behavioral emergencies are the second most common cause of EMS activation for patients less than 18 years old [5].

In contrast with adult BHE, pediatric BHE patients may have unique neurodevelopmental conditions and needs which may not be addressed in adult EMS protocols or in routine EMS clinician training. The increase in prehospital pediatric BHE also presents an operational and clinical stress point for EMS agencies and clinicians. Given the overall rise in pediatric BHE, evidence-based guidelines (EBGs) and/or evidence-based protocols (EBP) for the prehospital management of pediatric BHE may help optimize patient outcomes and EMS clinician safety. However, to date, an EBG for pediatric BHE has not been developed and is needed in the US. Therefore, the objective of this study was to perform a scoping review of published research related to the prehospital management of pediatric BHE and an internet search of publicly available statewide EMS pediatric BHE protocols or guidelines.

## Review

Materials and methods

Scoping Review Search & Selection Criteria

We searched the published research literature using strategies created by a medical librarian (CA) and other authors (EZ, JF) for papers relevant to pediatric BHE in the prehospital setting. The search was performed in PubMed and Google™ for English language manuscripts published between January 1, 2012 and May 1, 2022. That date range was chosen because of the supporting literature demonstrating a rising incidence of pediatric BHE within the last decade [5]. The search strategy used a combination of standardized terms and keywords, including, but not limited to the following boolean search terms: (pediatrics OR pediatric OR child) AND (behavioral emergencies OR agitation OR sedation OR de-escalation) AND (emergency treatment OR emergency department OR pediatric emergency department OR prehospital OR paramedic). The age range of patients included in the studies was set at zero to 21 years old in concordance with the American Academy of Pediatrics' definition of pediatric age ranges [6]. One author (EZ) initially screened the titles and abstracts of all identified papers for inclusion. Those papers were then reviewed by authors EZ, JF, and KK for final inclusion. Original research studies describing the incidence and epidemiology of pediatric behavioral emergencies and inclusion of either prehospital and/or in-hospital interventions were included. Papers from the ED setting were included in this review as many patients arrive in the ED via EMS. 

To search for publicly available statewide EMS protocols/guidelines, (protocols refer to medical director-authorized treatment protocols, while guidelines refer to suggestions for items to incorporate or consider in protocols), a Google™ search was performed in November 2020 by one author (LM) to identify U.S. (United States) state EMS protocols related to the management of pediatric BHE and/or agitated pediatric patients. The decision was made to search for US state protocols as they may be more likely publicly available on the internet in contrast to local agency protocols. Key search terms included but were not limited to pediatric agitation, restraints, and the Stabilization, Acknowledgment, Facilitate Understanding, Encouragement, Recovery/Referral (SAFER) method [7]. Forty-three state EMS protocols were manually screened by one author (LM) for either pediatric-specific BHE protocols or an adult BHE protocol that contained pediatric-specific elements.

Major findings of the research studies and specific management actions identified in the review of the EMS protocols/guidelines were abstracted and summarized (EZ). For research studies, information related to publication year, setting, patient characteristics, and interventions were abstracted. For EMS protocols, the protocol/guideline source, and procedure and medication recommendations were abstracted. All authors then reviewed the preliminary findings.

Results

**Figure 1:**
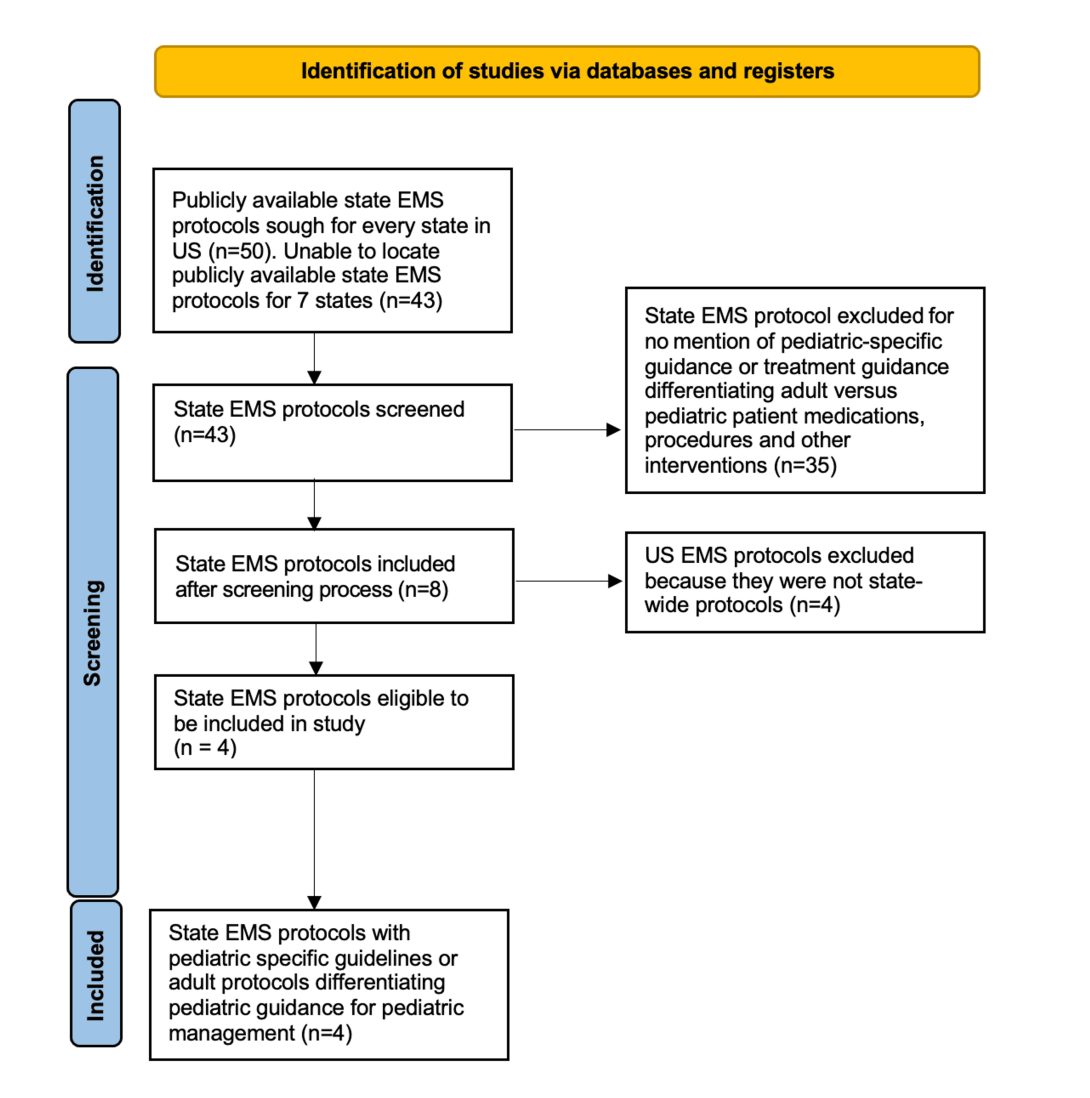
Identification of US EMS protocols for inclusion criteria. EMS- Emergency Medical Services

**Figure 2:**
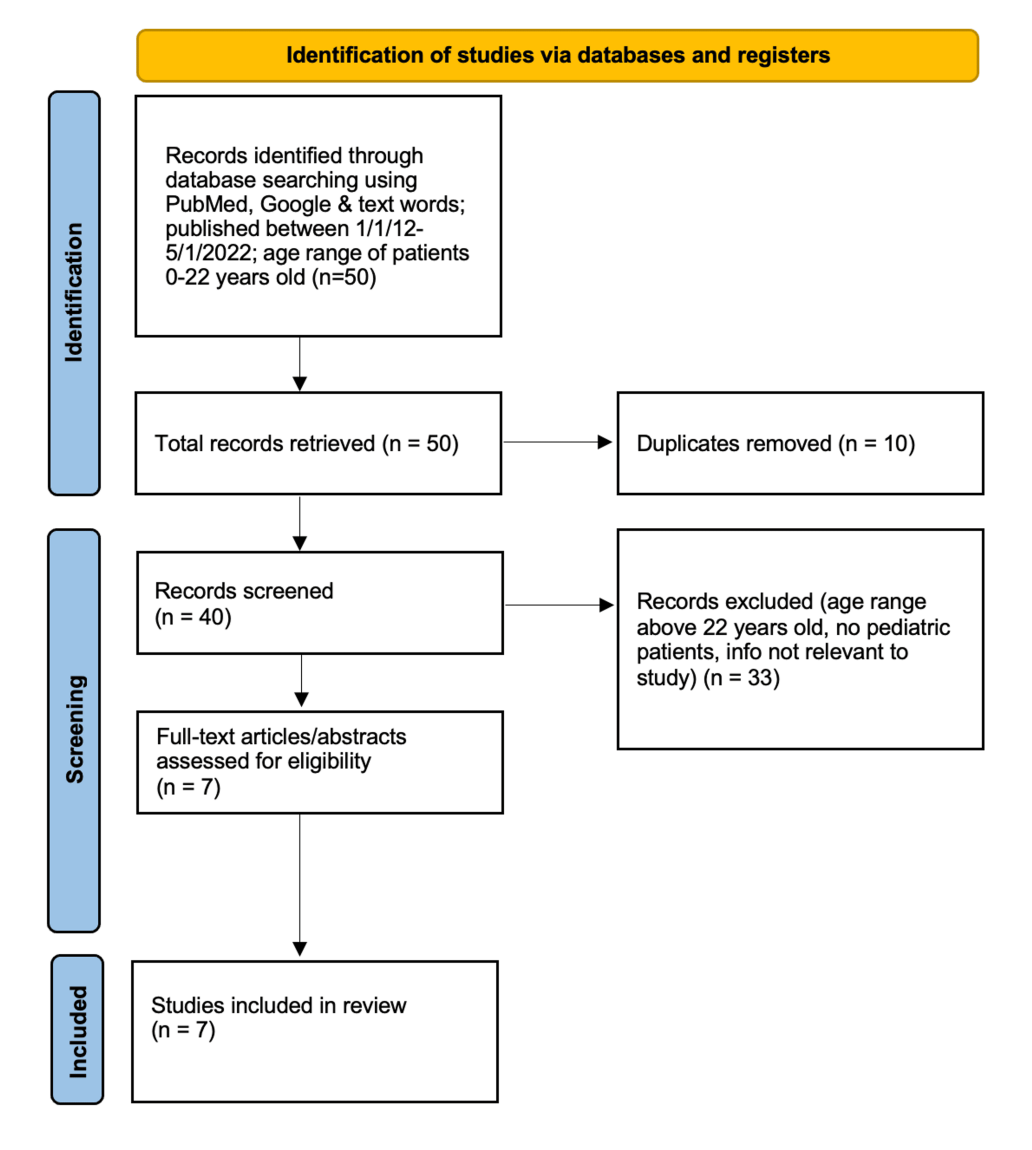
Identification of research studies for inclusion criteria

This scoping review identified a total of 50 research studies and 43 EMS protocols for screening, of which a total of seven research studies and four state EMS protocols were included for in-depth analysis (Figures 1-2). Of the seven research papers included, three discussed the rising incidence of pediatric BHE in the prehospital and ED settings [8-10] (Table 1). Two of those three articles [8,10] also show supporting evidence for an increased incidence of pediatric BHE during and after the COVID-19 pandemic.

**Table 1:** Included research publications related to rising incidence of pediatric behavioral health emergencies. PED - Pediatric Emergency Department, BHE- Behavioral Health Emergency, COVID-19- Coronavirus disease 2019

	Title	Authors	Publication Year; Journal; Country	Type of Publication & Study	Findings
1.	Report finds pediatric behavioral health emergencies on the rise, accelerated by pandemic	Barnhart C [8]	Journal of Emergency Medical Services; 4/22/22; USA	Summary report with ImageTrend Collaborate research and analysis program data	Over 1 million pediatric BHEs in the prehospital setting from 2018-2022. 11% (111,079) of incidents were pediatric BHE. There was a 10% increase in pediatric BHE from 2018-2021 There was a 19% increase in pediatric BHE one year after the COVID-19 pandemic began.
2	Trends in pediatric emergency department utilization for mental health-related visits	Mapelli et al. [9]	The Journal of Pediatrics; 7/1/2015; Canada	A retrospective cohort study of tertiary pediatric emergency department (PED) visits from 2003-2012. All visits with chief complaints or discharge diagnoses related to mental health were included.	Observed a 47% increase in the number of mental health presentations compared with a 9% increase in the number of total visits to the PED over the study period.
3	Mental health-related visits in a pediatric emergency department during the COVID-19 pandemic	Fernandez et al. [10]	International Journal of Emergency Medicine; 11/2/2021; France	Epidemiologic study of all PED visits (focusing on mental health-related visits versus total visits) at the University Children’s Hospital of Nice from January 2017-December 2020.	From January 1, 2020 - December 31, 2020 (the year of the COVID-19 pandemic), there was a 44.2% increase in visits pertaining to pediatric mental health when compared to January 1,2017-December 31, 2019. During this time, there was also a 30% decrease in total visits to the PED.

The remaining four research publications included in this review discussed prehospital interventions for pediatric BHE patients (Table 2). Those articles included a case study, a prospective study, a retrospective study, and a systematic review. The case study described the successful de-escalation and use of chemical restraint of a pediatric patient with autism spectrum disorder using diphenhydramine and droperidol [11]. The prospective study by Page et al. discussed the safety and effectiveness of droperidol for pediatric BHE patients concluding a low risk for life-threatening adverse events and spontaneous resolution of minor adverse effects [12]. Out of 102 patients, there were nine adverse effects in eight patients (hypotension, dystonic reactions, and respiratory depression). Among the five patients with hypotension, all were asymptomatic, and only one required intravenous fluids. There were two dystonic reactions managed with benztropine and one patient with respiratory depression that was monitored with symptoms resolving spontaneously. The retrospective study by Fishe and Lynch was a statewide study from Florida and reported 22,254 pediatric BHE-related EMS encounters from 2011-2016 [13]. Notably, 25% of those patients had suspected or confirmed ingestions, and 4% required EMS intervention. Those interventions included medications and physical restraints. Naloxone was the most frequently administered prehospital medication and midazolam was the most frequently administered sedative [13]. The last article was a 2022 systematic review (by Ramsden et al.) based in the United States that looked at the effectiveness and safety of droperidol for pediatric agitation in acute care settings [14]. Overall, the review found that the existing data on droperidol for the management of acute agitation in children suggested that droperidol is both effective and safe for acute, severe agitation in children [14].

**Table 2:** Included research publications regarding prehospital interventions for pediatric behavioral health emergency patients. IM- Intramuscular, SBP- Systolic Blood Pressure

	Title	Authors	Publication Year & Journal	Ages Included	Sample Size	Primary Outcome	Medications Examined
1	Prehospital chemical restrain of a noncommunicative autistic minor by law enforcement	Ho J et al. [11]	2012, Prehospital Emergency Care, US	16 yo M	1 patient	A case study demonstrating successful use of chemical restraint in the prehospital setting.	IM Droperidol and diphenhydramine
2	A prospective study of the safety and effectiveness of droperidol in children for prehospital acute behavioral disturbance	Page et al. [12]	2019, Prehospital Emergency Care, Australia	< 16 years	102 presentations in 96 patients	The proportion of adverse effects included the need for airway intervention, oxygen saturation < 90%, RR < 12, SBP < 90, sedation assessment tool < -3, and dystonic reaction.	IM droperidol
3	Pediatric behavioral health-related EMS encounters: A statewide analysis	Fishe et. al. [13]	2019, Prehospital Emergency Care, US	2-18 years	22,254 EMS encounters	Retrospective study of pediatric behavioral health-related EMS encounters from Florida’s statewide EMS tracking and Reporting Systems Database from 2011-2016. Only 4% of these patients required interventions from EMS. The most utilized medication was naloxone, which correlates with the high number of ingestions/suspected ingestions within this population. Twenty-six percent of patients required physical restraints and 24% required “psychological first aid”.	Use of Naloxone for ingestion and use of Midazolam for sedation
4	A systematic review of the effectiveness and safety of droperidol for pediatric agitation in acute care settings	Ramsden et al. [14]	2022, Academy of Emergency Medicine, US	< 21 years	431 articles identified; 6 articles met inclusion criteria; 2 prehospital settings, 1 in the ED, 3 inpatient setting	Data suggests that droperidol is effective and safe for acute severe agitation in children. The median time to sedation was 14 minutes. 8-22% of patients required a second dose of medication. The most frequent adverse effects were dystonic reactions and transient hypotension.	Parenteral Droperidol

Summaries of two states with publicly available pediatric-specific state EMS BHE protocols (Maryland [15] and Utah [16] ), and two states with pediatric-specific recommendations included in an adult excited delirium protocol (Michigan [17] and Rhode Island [18] ) are shown in Table 3. All protocols emphasize the need to establish scene safety, exclude other medical emergencies/causes of altered mental status, and use non-pharmaceutic de-escalation techniques prior to the administration of medications. However, only one protocol (Utah) gave examples of de-escalation techniques. The medications included in the four protocols were ketamine, midazolam, and haloperidol.

**Table 3:** State emergency medical service protocols included in this review with provided details. EMS- Emergency Medical Services, EMT- Emergency Medical Technician

State	State Protocol Title	Pediatric-specific protocol or adult protocol with pediatric recommendations	Protocol details	Most utilized medications
Maryland	The Maryland medical protocols for EMS 2020 [15]	Pediatric-specific	Protocol for “Excited Delirium Syndrome” with a separate section on administering chemical restraints to agitated pediatric patients. No mention of pediatric patients in the “Behavioral Emergencies” Section. This section discusses the SAFER model and advises considering chemical constraints.	Patients less than 13 years old require consultation with a physician before the use of chemical restraint. Recommends ketamine for patients 13-18 years old.
Utah	Utah EMS protocol guidelines 2020 [16]	Pediatric-specific protocol	Protocol for “Altered Mental Status” has separate guidelines for pediatric patients less than 15 years old. “Violent/Chemical Sedation/Taser Barb Removal” has specific pediatric recommendations for chemical restraint. Advises chemical restraint should be considered for patients “that cannot be calmed by non-pharmacologic methods and are dangers to themselves and EMS.” Gives examples such as “attempt to calm or gently redirect patient with verbal reassurance. Engage the assistance of any family members present in the process.”	Recommendations based on the level of EMS clinician on scene. For Advanced Emergency Medical Technicians (AEMT) midazolam, diazepam and lorazepam are advised. For paramedics, ketamine and haloperidol are advised.
Michigan	Michigan state protocols bureau of EMS, trauma and preparedness general treatment protocols [17]	Adult protocols with pediatric recommendations	Protocols for “Excited Delirium,” and “Behavioral Health Emergencies” recommend “communicate in a calm and nonthreatening manner, attempt de-escalation and utilize an empathetic approach. Instructions for chemical restraints are located in “Medication Selection” (section 9-1, pages 1-2), with specific recommendations for pediatric sedation.	Recommends weight-based dose of midazolam for "pediatric sedation”.
Rhode Island	Rhode Island statewide emergency medical services protocols 2020 [18]	Adult Protocols with pediatric recommendations	Includes protocol for “Excited Delirium” and “Behavioral Health Emergencies” with chemical restraint recommendations for patients 16 years and older. No recommendations or mention of pediatric patients less than 16 years old in either protocol	Prefers the use of ketamine but also recommends midazolam, and haloperidol.

Discussion

The incidence of pediatric BHE is rising in the United States. Despite this, we found only a few published research papers and publicly available state EMS protocols to guide the prehospital management of pediatric BHE patients in this scoping review. Additionally, there appears to be a paucity of research or EMS protocol considerations for children with neurodevelopmental conditions.

Over a 10-year period, we found only seven articles that directly relate to pediatric BHE in the out-of-hospital setting. Of note, the published literature ranged in quality of evidence from very low (case report) to moderate (prospective study). Of the seven studies, two discussed the safety and efficacy of droperidol, whereas published EMS protocols contain ketamine, midazolam, diazepam, and haloperidol. Thus, there may be an opportunity for consolidation of sedation medication options within EMS protocols and an opportunity for further research looking at the prehospital use of droperidol in comparison to other sedation medications currently used for pediatric BHE patients. Of note, the use of droperidol greatly decreased in 2001 after its black box warning for QTc prolongation but was reintroduced in the US in 2019 after clarification that its black box warning applied to doses of droperidol that are greater than 2.5 milligrams (mg) [19]. Interestingly, the two included studies in this scoping review that utilized droperidol used doses of 10 mg [12,13]. In 2021 the American College of Emergency Physicians (ACEP) issued a policy statement reaffirming the safety and efficacy of droperidol for a variety of common conditions treated in adults including headache, nausea, vomiting, and agitation, and recommended droperidol for the ED and out-of-hospital management of agitated psychosis in doses up to 20 mg provided that cardiac monitoring is available soon after administration [20]. However, the policy statement failed to include recommendations for children and adolescents. Thus, further research on the safety and monitoring required for droperidol use in pediatric prehospital patients is needed.

With regard to assessment and appropriate monitoring, the study by Page et al. discussed the use of a sedation assessment tool (SAT) before and after giving pharmaceutical treatment for agitation [12]. Studies have shown that this scale is a useful measure of the level of agitation in patients and can also be used to score the level of sedation of patients after chemical restraints are administered in the emergency department setting and allow clinicians to titrate the effect [21]. This original study by Calver et al. discussing SAT does not include pediatric patients [21]. Therefore, given the developmental differences between adults and children, the application to pediatric patients may not be appropriate. Moreover, the development and use of other sedation assessment tools in the prehospital setting would also be important to inform the development of an evidence-based management guideline for prehospital pediatric BHE patients.

One case study of this review demonstrates the importance and challenges of pediatric neurodevelopmental conditions in the management of pediatric BHE. Autism spectrum disorder (ASD) is not unique to the pediatric population; however, children with ASD often have behavioral health emergencies. Patients with ASD and other neurodevelopmental conditions can have increased pain tolerance, inability to express pain, inability to communicate expressively, and impaired receptive communication. These factors can hamper the prehospital assessment, management, and transport of a pediatric patient with ASD or other neurodevelopmental conditions, especially when a caregiver is not available to assert the patient’s baseline behavior and attest to any home medications that may also cause agitation [22]. Given that the literature on this condition is limited to one case study, further research is needed to identify if there should be EMS protocol considerations for children with neurodevelopmental conditions.

In this study, we only identified four states with state protocols that addressed pediatric BHE. Interestingly, all of the included EMS protocols contained common themes including the importance of addressing medical emergencies that can cause agitation or altered mental status, and situational “de-escalation” before the use of medications. The corresponding research literature in this scoping review did not contain evidence for various types of non-pharmacologic de-escalation techniques. Research on the identification and examination of non-pharmaceutical de-escalation techniques in the prehospital setting is needed. Non-pharmacologic techniques are especially impactful for EMS providers who are unable to administer medications in the prehospital setting.

This study has limitations that merit consideration. The quality of the evidence of this scoping review is variable and our search was limited to only 10 years. Older studies may have provided evidence for management strategies in pediatric BHE missed in our date range of literature review. As in systematic reviews, this study may be subject to publication bias as well. In addition, the review of publicly available state EMS protocols for pediatric BHE is not a complete review of all US EMS protocols, as not all states have statewide EMS protocols. Therefore, it is most likely that there are local/regional EMS protocols that contain pediatric-specific BHE recommendations.

## Conclusions

Although there is a rise in pediatric BHE, there is sparse research data to support prehospital EMS practices and protocols. There is also a notable absence of literature regarding more pediatric-specific neurodevelopmental conditions that can also exhibit behavioral health issues and non-pharmacologic techniques for EMS to incorporate into the assessment and management of prehospital pediatric BHE. What little evidence does exist suggests that drugs such as droperidol could be considered for incorporation into EMS protocols for chemical sedation when non-pharmacologic methods fail. Given the rising incidence of pediatric BHE, research and clinical quality assurance must be performed and results expeditiously disseminated to support EMS agencies and EMS clinicians to optimize patient outcomes and provider safety.

## References

[1] Lo CB, Bridge JA, Shi J, Ludwig L, Stanley RM (2020). Children's mental health emergency department visits: 2007-2016. Pediatrics.

[2] (2023). AAP- AACAP-CHA declaration of a national emergency in child and adolescent mental health. https://www.aap.org/en/advocacy/child-and-adolescent-healthy-mental-development/aap-aacap-cha-declaration-of-a-national-emergency-in-child-and-adolescent-mental-health/.

[3] Miller ML, Lincoln EW, Brown LH (2021). Development of a binary end-of-event outcome indicator for the NEMSIS Public Release Research Dataset. Prehosp Emerg Care.

[4] (2023). Pediatric behavioral health incidents in the prehospital setting from 2018 to 2021. https://assets.cdnma.com/13864/assets/Collaborate%20Report/National%20Short%20Report_Collaborate-Ped%20Behavioral_FEB%202022.pdf.

[5] Lerner EB, Browne LR, Studnek JR (2022). A novel use of NEMSIS to create a PECARN-specific EMS patient registry. Prehosp Emerg Care.

[6] Hardin AP, Hackell JM (2017). Age limit of pediatrics. Pediatrics.

[7] Wang D, Gupta V (2023). Crisis Intervention. NCBI Bookshelf- StatPearls.

[8] Barnhart C. (2023). Report finds pediatric behavioral health emergencies on the rise, accelerated by pandemic. https://www.jems.com/patient-care/report-finds-pediatric-behavioral-health-emergencies-on-the-rise-accelerated-by-pandemic/.

[9] Mapelli E, Black T, Doan Q (2015). Trends in pediatric emergency department utilization for mental health-related visits. J Pediatr.

[10] Fernandez A, Gindt M, Babe P, Askenazy F (2021). Mental health-related visits in a pediatric emergency department during the COVID-19 pandemic. Int J Emerg Med.

[11] Ho JD, Nystrom PC, Calvo DV, Berris MS, Norlin JF, Clinton JE (2012). Prehospital chemical restraint of a noncommunicative autistic minor by law enforcement. Prehosp Emerg Care.

[12] Page CB, Parker LE, Rashford SJ, Isoardi KZ, Isbister GK (2019). A prospective study of the safety and effectiveness of droperidol in children for prehospital acute behavioral disturbance. Prehosp Emerg Care.

[13] Fishe JN, Lynch S (2019). Pediatric behavioral health-related EMS encounters: a statewide analysis. Prehosp Emerg Care.

[14] Ramsden SC, Pergjika A, Janssen AC, Mudahar S, Fawcett A, Walkup JT, Hoffmann JA (2022). A systematic review of the effectiveness and safety of droperidol for pediatric agitation in acute care settings. Acad Emerg Med.

[15] (2022). The Maryland medical protocols for emergency medical services providers. http://www.miemss.org/home/Portals/0/Docs/Guidelines_Protocols/MD-Medical-Protocols-2018-WEB.pdf.

[16] (2023). Utah EMS protocol guidelines: general. https://www.uvu.edu/ert/docs/2017-uepg-general.pdf.

[17] (2023). Michigan general treatment: general pre-hospital care. https://www.michigan.gov/documents/mdhhs/Section_1_General_Treatment_Updated_3.23.2018_618637_7.pdf.

[18] (2022). Rhode Island statewide emergency medical services protocols. https://health.ri.gov/publications/protocols/StatewideEmergencyMedicalServices.pdf.

[19] Kramer KJ (2020). The surprising re-emergence of droperidol. Anesth Prog.

[20] (2022). Use of droperidol in the emergency department- American College of Emergency Physicians policy statement. https://www.acep.org/globalassets/new-pdfs/policy-statements/use-of-droperidol-in-the-emergency-department.pdf.

[21] Calver LA, Stokes B, Isbister GK (2011). Sedation assessment tool to score acute behavioural disturbance in the emergency department. Emerg Med Australas.

[22] Frankel Frankel, Cynthia Cynthia, Brian Blaisch, and Bruce Hagen (2022). Meeting the challenges of pediatric behavioral emergencies. Meeting the Challenges of Pediatric Behavioral.

